# Biomimetic Growth of Calcium Oxalate Hydrates: Shape Development and Structures in Agar Gel Matrices

**DOI:** 10.1002/chem.202404269

**Published:** 2025-04-14

**Authors:** Annu Thomas, Paul Simon, Wilder Carrillo‐Cabrera, Elena Sturm

**Affiliations:** ^1^ Department of Chemistry Bishop Chulaparambil Memorial College Kottayam, Kerala 686001 India; ^2^ Max-Planck-Institut für Chemische Physik fester Stoffe Nöthnitzer Straße 40 01187 Dresden Germany; ^3^ Ludwig-Maximilians-Universität München Department für Geo- und Umweltwissenschaften Theresienstr. 41 80333 München Germany

**Keywords:** biomimetic synthesis, calcium oxalates, morphology, agar gel, double diffusion

## Abstract

Crystal growth of calcium oxalate hydrates (COM: calcium oxalate monohydrate; COD: ‐dihydrate; COT: ‐trihydrate) is a specific example of pathological biomineralization due to their harmful role as kidney/urinary stones. In this work, the biomimetic growth of calcium oxalate hydrates has been achieved using double diffusion technique in agar gel matrix. *In vitro* experimental models for the growth of calcium oxalates can give valuable information on the formation of biominerals of kidney/urinary stones. Diverse morphological forms of COM are grown in agar gel matrices ranging from platy crystallites to dumbbells and spherulites. The morphology of COM grown in agar gel resembles COM biominerals remarkably. Furthermore, it has been discovered that a higher pH of the agar gel promotes COD development while suppressing COM growth.

## Graphical abstract

The biomimetic growth of calcium oxalate hydrates is conducted using a double diffusion technique in agar gel. The results reveal the shape development of calcium oxalate monohydrate (COM) and dihydrate (COD), resembling biominerals of kidney stones. The pH variations influence phase selection, with COM forming at lower pH and COD at higher pH. The findings offer insights into pathological biomineralization and urolithiasis.

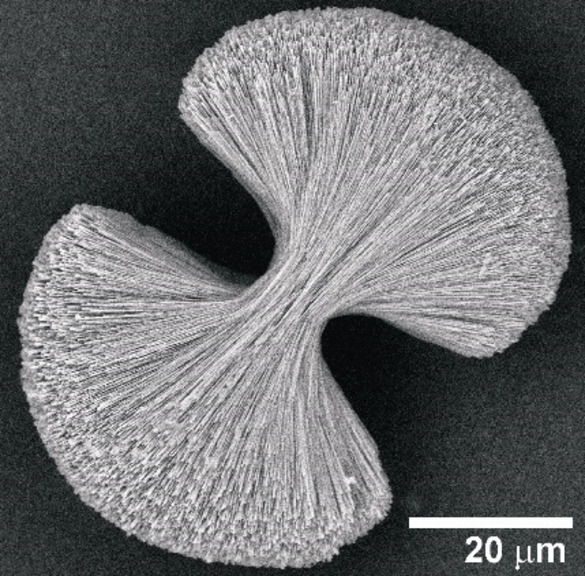

## Introduction

Investigating the growth of calcium oxalate hydrates (COM: calcium oxalate monohydrate; COD: ‐dihydrate; COT: ‐trihydrate) in the presence of organic additives is significant in the context of biomineralization principles. Though the biogenesis of calcium oxalates is pathological in humans,^[1]^ it is a normal physiological process in plants.^[2]^ While triclinic COT is not usually found in urinary concretions, COM and COD are the most prevalent phytocrystals and are among the principal components of kidney/urinary stones.

A variety of shapes, such as dumbbells or spherulites may be adopted by kidney/urinary calculi. These morphologies are created by the accumulation of smaller crystallites and “non‐crystallographic” branching.[^3^, ^4^] In addition to kidney, calcium oxalate crystals are seen in neutral and alkaline urine, where COM crystallizes into oval or dumbbell‐shaped aggregates whereas COD into tetragonal bipyramidal‐prismatic crystals.^[5]^ Calcium oxalate stones comprise of 3 to 5 weight percent of proteins, lipids, glycosaminoglycans, polysaccharides, and cellular debris.[^6^, ^7^] It is believed that the organic matter embedded within the calculi promotes aggregation and crystal adhesion to cells.[^8^, ^9^, ^10^] It was further hypothesized that, the accumulation of plate‐like COM crystallites with flat {−101} faces stacked one on top of the other results in the formation of dumbbell‐shaped COM.[^3^, ^11^] Aggregation of COM crystallites is a significant step in renal stone development.^[11]^ There is only a bare understanding about the precise nature of the link between the macromolecules and their inorganic components in calculogenesis, despite the substantial research that has been done. Growing calcium oxalates comparable in morphology to uroliths under similar growth conditions is crucial to comprehend this pathogenic biomineralization. The growth of calcium oxalate calculi within a gel‐like state of biomacromolecules with continuous urine flow provides evidence that an “organic” gel model can accurately represent the in‐vitro creation of urinary stones.^[12]^

It is typical to utilize the “double diffusion technique” with a gel matrix to grow crystals of substances with limited solubility.^[13]^ Studies conducted by Bisaillon and Tawashi^[14]^ and Frey‐Wyssling^[2]^ on the growth of calcium oxalate crystals in calfskin gelatin and agar gels showed that the morphology of the crystals are extremely sensitive to the growth conditions. The subsequent research concluded that COM development was feasible in synthetic gels like sodium metasilicate gel or bentonite clay.[^15^, ^16^] Follow up investigations to develop calcium oxalate crystals in a variety of gels, including gelatin, agar‐agar, agarose, and sodium silicate have shown twins, rosettes, and clusters of COM, but no signs of COD or COT.[^17^, ^18^, ^19^]

Attempts to grow calcium oxalates in gel systems, comparable in morphology to the biominerals and calculi have so far met with little success. We decided to investigate the intricate aetiology of uro/nephron‐ lithiasis by crystallizing calcium oxalates in organic gel matrices. Our previous investigations on the shape development of COD in the presence of increasing amounts of sodium salt of polyacrylic acid, produced a sequence of morphologies from flat bipyramids to bipyramidal prisms to dumbbells and finally to rod like tetragonal bipyramidal prisms.^[20]^ From atomistic simulations it was evident that the incorporation of PAA into the prismatic zones of the COD crystals, reduces the growth rate of {100} faces and increases defect density, which then gives rise to “non‐crystallographic” branching from the prism faces. This paper is the result of a series of investigations done with calcium oxalates growth in gelatin, agar, agarose and carrageenan gels in pursuit of a model system to get deeper insight into the growth and shape development of urinary stones.^[21]^ The present work focuses on the influence of agar gel on controlling the phase and morphology of calcium oxalates.

As biogenic calcium oxalates are formed in the presence of proteins and polysaccharides,[^7^, ^22^] a lot of work has been reported about *in‐vitro* stone genesis in the presence of polyelectrolytes and acid proteins, while polysaccharides are almost ignored.[^23^, ^24^, ^25^] Agar is used therefore, with an intention to mimic the urinary polysaccharides to understand the genuine function of polysaccharides on calcium oxalate biomineralization.

## Results and Discussion

**Figure 1:**
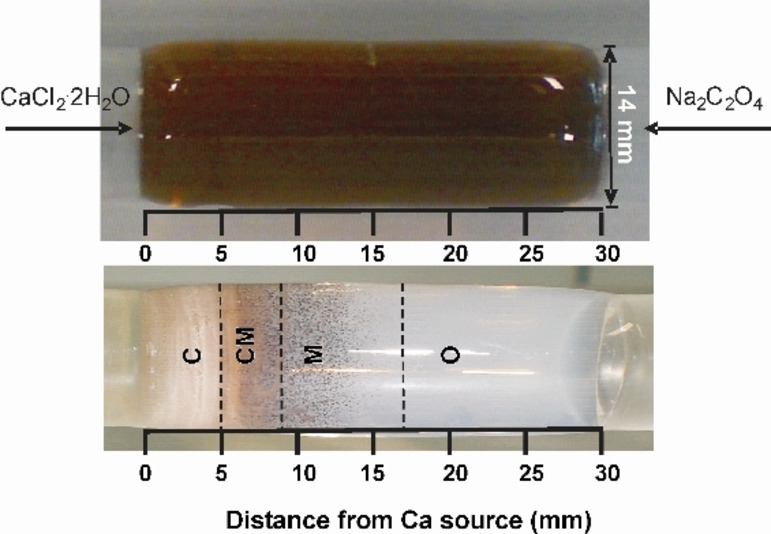
2 wt % agar gel of 30 mm length and pH 8.5: (Top) before the diffusion of calcium chloride and sodium oxalate solutions. (Bottom) The Liesegang band assignments in the same gel after the double diffusion reaction for 3 days.

Double diffusion experiments were conducted in 2 wt % agar gel of pH values 5, 8.5 and 11.5 at 37 °C. The calcium oxalate aggregates were formed inside the agar gel matrix in periodically arranged bands called Liesegang bands over a period of 3 days. The bands were named according to the distance from the calcium source as shown in Figure 1. The band, located within 5 mm from the calcium source is named C and the one close to the oxalate source which is within a distance of 17 to 30 mm from the calcium source is named O. The CM and M (middle) bands are located within distances 5 to 9 and 9 to 17 mm from the calcium source, respectively.

**Figure 2:**
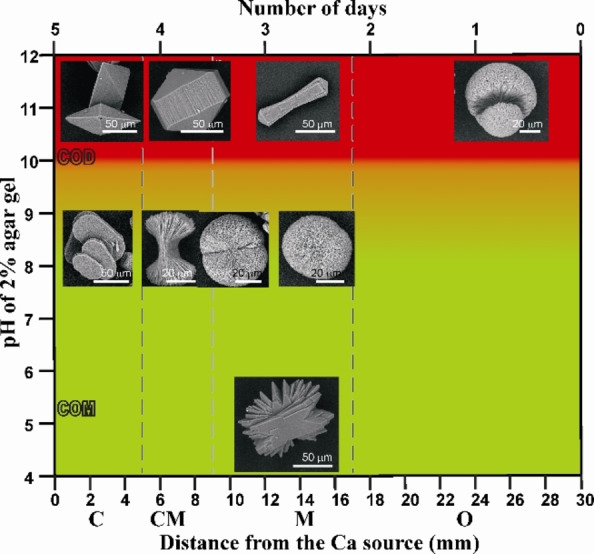
Calcium oxalate aggregates formed in 2 wt % agar gels at various pH values: morphology and hydration state map. The results are shown as a function of the pH of the gel, the location of the aggregates inside the gel and the number of days after which the crystals were grown inside it. The green area of the map indicates the occurrence of COM and red area, COD.

It had been noted that a change in the pH value of the gel had an impact on the hydration state and the morphology of calcium oxalates formed. COM was formed at gel pH values <9 and COD crystals were dominant at gel pH values >10. The summary of results obtained in agar gel as a function of pH of the gel and the location of the aggregates inside the gel are depicted in the map (Figure 2). At pH 5, COM crystals formed were rosette‐type at the exact middle of the gel. Different morphologies of COM, including spherulites and dumbbells, were developed when the pH of the gel was raised to 8.5. At higher pH values (11 to 12), tetragonal bipyramidal‐prisms and dumbbells of COD were formed (See Figure S1 and S2 for the pictures of gels before and after the diffusion).

**Figure 3:**
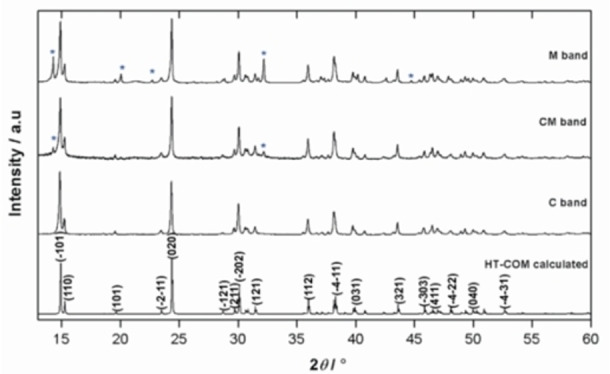
XRD patterns of the aggregates formed in Liesegang bands of 2 wt % agar gel of pH 8.5 at 37 °C in comparison with the calculated pattern for HT‐COM.[^26^, ^27^] The data was measured using Cu *K*α_1_‐radiation.

According to XRD patterns, the aggregates generated in the M, CM, and C bands of a 2 wt % percent agar gel at a pH of 8.5 correlate to the high temperature modification of COM, HT‐COM (Figure 3).[^26^, ^27^]

The commonly accepted crystal structure of COM was performed by Tazzoli and Domeneghetti.^[27]^ COM also exists in two phases that transform reversibly in the temperature range 38 to 45 °C. Both have monoclinic structures and are designated as high temperature (HT) structure (stability range ca. 45 to 152 °C) and the low temperature (LT) structure (stability range ca. 20 to 45 °C).^[26]^ The scattered M band was formed after two days and consisted of COM spherulites (major) and COD dendrites (minor). The fraction of COD in the M band has been calculated from the major XRD peak intensities as 36.57(7) %. The CM band formed after 3 days, adjacent to the middle band, but more towards the Ca^2+^ reservoir, consisting of mainly dumbbell shaped COM. The C band is comprised of twinned crystals of COM. The order of appearance of these bands in the present study followed the order: M bands were formed first, followed by CM bands and C bands were formed last.

The fraction of COD formed (reflections from COD are marked with *) increases on moving from C to M band. The calculated fraction of COD in the CM and M bands are approximately 29.25 % and 36.57 % respectively. Hence, the M band which is formed earlier than CM and C bands have more fraction of COD aggregates.

**Figure 4:**
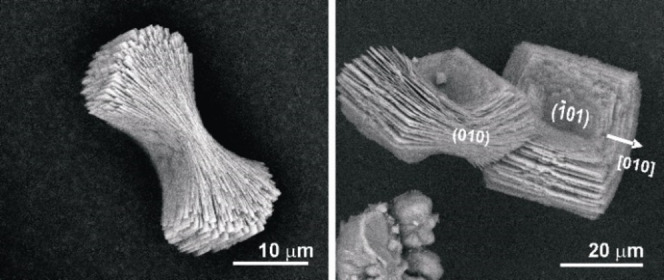
(Left) SEM image of a COM aggregate having dumbbell/sheaf of wheat morphology formed in the CM band of 2 wt % agar gel of pH 8.5. (Right) The presence of rectangular plate‐like crystals with dominant {−101} faces is evident. The view along $\left\langle 010\right\rangle$ gives the dumbbell morphology.

Figure 4 represents the shape of the COM aggregates formed in the CM bands. Due to the arrangement of roughly rectangular plate‐like crystallites that are overgrown, one on top of the other, due to the “non‐crystallographic” branching, these aggregates have a general dumbbell shape (or sheaf of wheat morphology).

**Figure 5:**
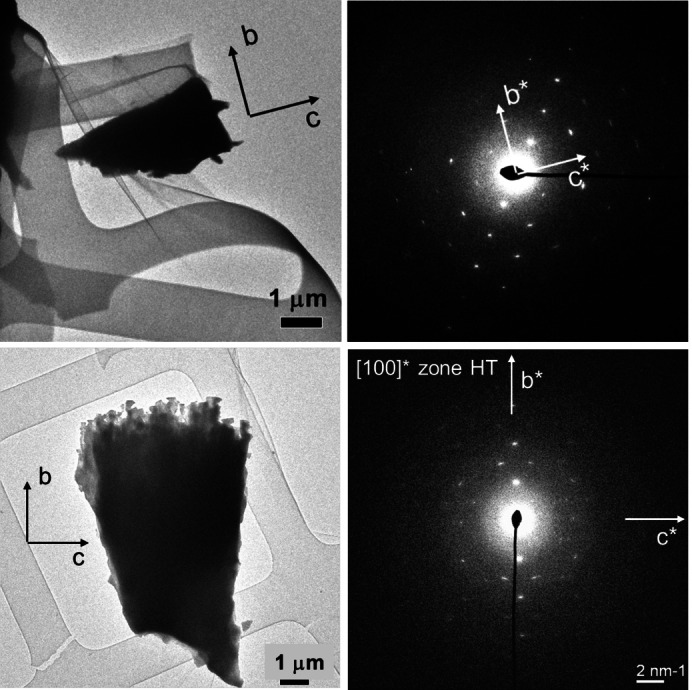
(Left) TEM images of fragments of a single plate detached from a COM dumbbell and (right) the corresponding electron diffraction patterns from an approximate [100] zone axis. These plates correspond to HT‐COM with monoclinic cell, *a*~10.0 Å, *b*~7.3 Å, *c*~6.3 Å, *β*~107° and space group *I*2/*m*.

A mechanically detached plate was examined by TEM to determine the crystallographic orientation of the COM plates. The electron diffraction pattern (ED) oriented approximately along the [100] zone axis corresponds to monoclinic COM with lattice parameters, *a*=10 Å, *b*=7.3 Å, *c*=6.3 Å, *β*=107 ° with space group *I*2/*m* which corresponds to the high temperature (basic) phase of COM (Figure 5). These plates are therefore (−101) dominating plates that are extended along the *a*‐axis. The view along [010] produces the dumbbell shape.

The “six‐sided” platy morphology of COM crystals are usually produced in high ionic strength solutions which typically includes three face types, {−101}, {010} and {−120}.^[9]^ It is interesting to note the morphology of the single COM crystal that makes up the dumbbell has been transformed from the typical “six‐sided” platy to a “rectangular” morphology with serrated edges when agar is present. These distinct rectangular plate‐like crystals possess well‐developed (−101) faces but essentially no apical planes (120) faces.

**Figure 6:**
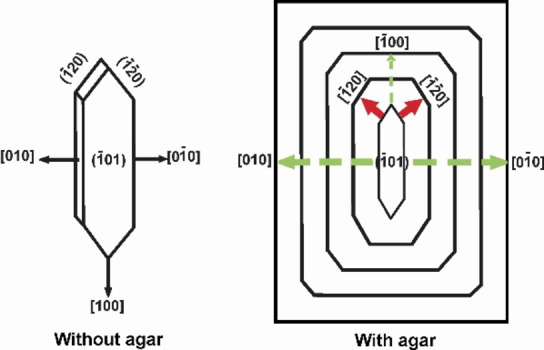
Habit of COM crystal in the absence (left) and in the presence of agar (right) depicting the transformation of the morphology from six‐sided platelet to rectangular platelet. Adsorption of the macromolecule on the {−101} planes results in the disappearance of the fast‐growing apical planes (red arrow). Slower growth of the {−101} faces results in increased surface area (green arrow) and preferential growth along $\left\langle 010\right\rangle$ .^[34]^

Comparable to agar, glycosaminoglycans and several dicarboxylates (malonate, malate, and maleate) have been reported to change the morphology of COM from a six‐sided plate to a rectangular plate.[^28^, ^29^] Also, COM grown in the presence of nephrocalcin has been reported to undergo a phase change to high temperature form with increase in concentration of nephrocalcin.^[30]^ The explanation for such a morphological change is that the macromolecule adsorbs on the {−101} planes, thus inhibiting crystal growth in the direction perpendicular to that face. Hence the {−101} faces shows an increased surface area by growing along [010] (Figure 6). It has been demonstrated by AFM measurements that the {−101} faces of COM and the {100} faces of COD exhibit maximum adhesion forces with the macromolecules.[^31^, ^32^] As a result, extended {−101} faces of COM adhere strongly to biological macromolecules and cell membranes surfaces.[^9^, ^33^]

As mentioned in the above cases, it is worth to assume that agar interacts with the {−101} faces of COM and blocks their growth. As a result, the {−101} faces are preferentially developed by its growth along $\left\langle 010\right\rangle$ (Figure 6). It has been proven that the apical planes ({120} faces) of COM are the fastest growing and the {−101} faces are the slowest.^[34]^ Due to the faster growth rate inside agar gel, the fast growing faces (apical planes) are eventually suppressed. The agar adsorbed on the {−101} crystal faces may initiate new crystal layers, similar to how the protein perlucin initiates new layers on geological calcite, which may explain the piling up of plates.^[35]^

**Figure 7:**
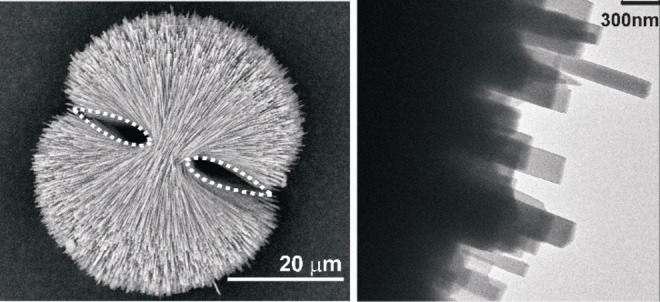
(Left) SEM image of COM grown from the CM band of 2 wt % agar gel of pH 8.5. The “double‐ eye” is evident (highlighted with white dots). (Right) TEM image of the outer crystallites indicating the rectangular shape of individual crystallites.

**Figure 8:**
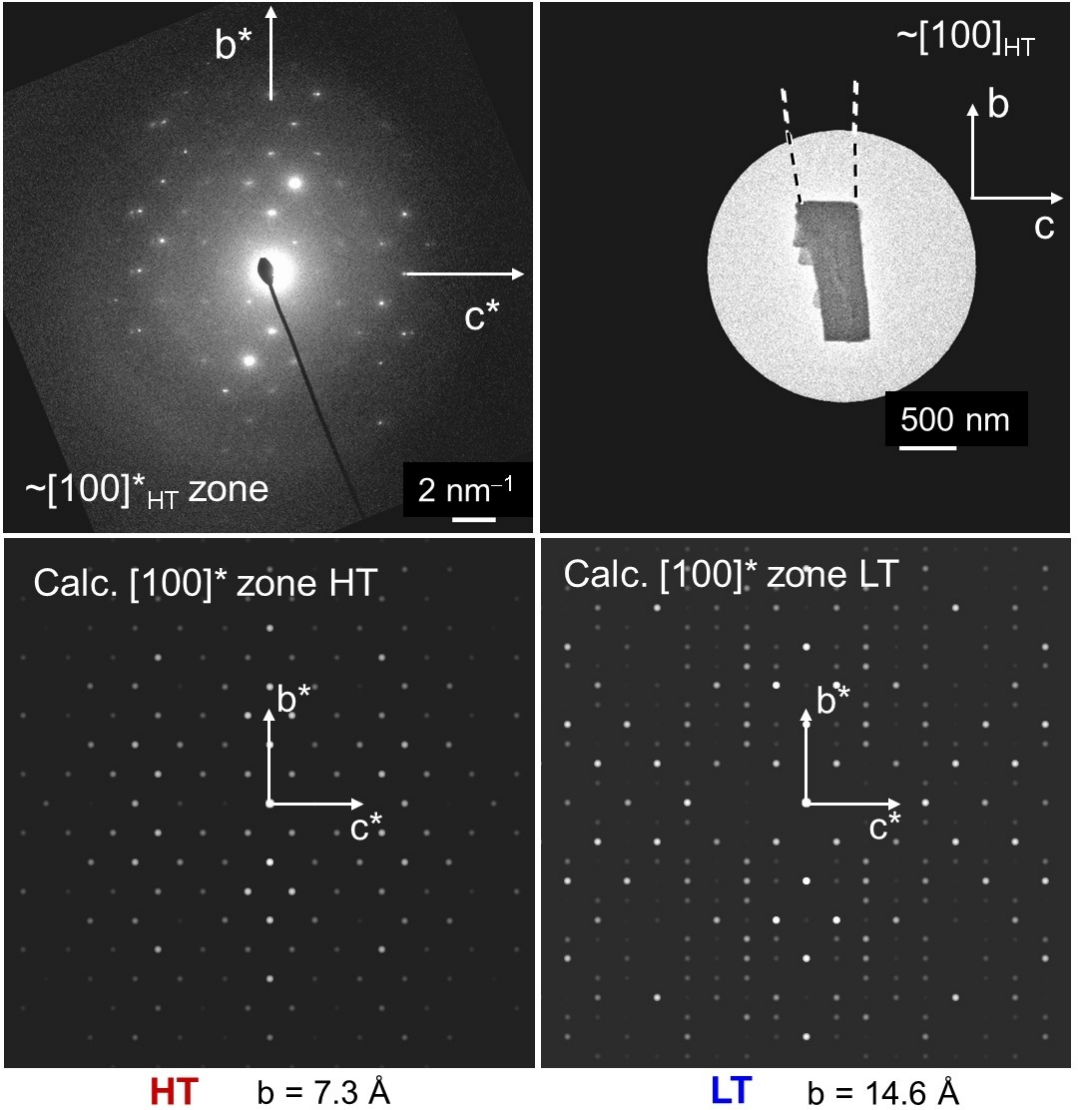
(Top left) Electron diffraction pattern from [100] zone axis and (top right) corresponding broken fragment of platy crystallite detached from the COM dumbbell obtained from the M band of 2 wt % agar gel of pH 8.5. This indicates the occurrence HT phase of COM. At the bottom simulated patterns of HT and LT phases of COM are shown.

The end faces of individual platy crystallites appear almost to touch (intergrow) each other to produce flattened “two‐dimensional” (2D) spherulites with the so called “double‐eye” in the case of COM aggregates grown in the CM band, but more towards the M band, (Figure 7). TEM examinations of such a highly branched species (Figure 7 right) confirmed that the individual crystallites are still rectangular shaped but more needle‐like than platy. Additionally, it was found from the electron diffraction (ED) pattern that the rectangular needle‐like crystals belong to the HT modification of COM (Figure 8).

**Figure 9:**
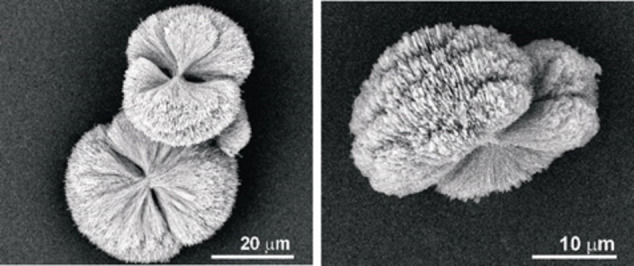
SEM images of COM grown from the CM band of 2 wt % agar gel of pH 8.5. Rectangular needle‐like crystallites are aggregated on both sides of an already formed dumbbell.

The needle‐like rectangular crystallites in the M band were formed faster than the CM band flatter rectangular plates as the calcium oxalate supersaturation was higher in the M band. These highly branched aggregates with needle‐like rectangular crystallites (subindividuals) result in an overall spherulitic shape. The rectangular plate‐like crystallites have an approximate aspect ratio (ratio of length along [100] to width along [010]) of 5 : 1 while that of rectangular needles is *ca*. 13 : 1, which is a consequence of increased supersaturation of the system and faster growth.^[36]^ From Figure 9 it is evident that after the formation of a 2D dumbbell, 3D shape development starts by formation of new subindividuals on the crystallites of the first formed bundle. The curved layer by layer appearance of the stacks confirms this fact. In the 2D dumbbell, if the crystal growth of {−101} is inhibited by the adsorption of agar the growth could take place preferably along $\left\langle 010\right\rangle$ and $\left\langle 100\right\rangle$ . In the case of needle‐like crystals, the aspect ratio is enhanced which proves the fact that the crystal growth along $\left\langle 010\right\rangle$ is retarded, and the crystal grows as much as it can along $\left\langle 100\right\rangle$ direction. At initial higher supersaturations the crystal growth along both $\left\langle -101\right\rangle$ and $\left\langle 010\right\rangle$ is hindered. This is possible when agar is adsorbed on {010} and {−101} faces.

**Figure 10:**
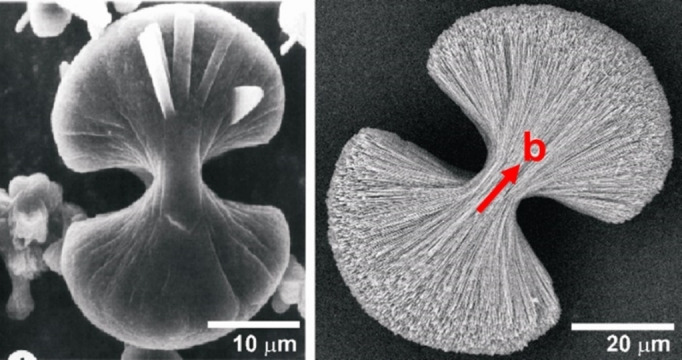
(Left) SEM image of a dumbbell shaped COM formed under *in vivo* conditions by glyoxylate intoxication in rats.^[9]^ (Right) Biomimetically grown COM from the CM band of 2 wt % agar gel of pH 8.5 at 37 °C.

The dumbbells biomimetically grown in the CM band of agar gel and the COM dumbbells found in human crystalluria resemble closely.[^5^, ^33^] The dumbbell shaped COM is commonly found in the urine of individuals suffering from intoxication with ethylene glycol, a metabolic precursor of oxalate, and in mice fed with glyoxylate (Figure 10).[^9^, ^37^]

**Figure 11:**
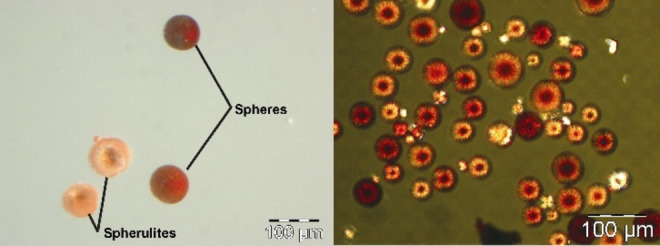
Light microscope images of (left) COM spherulites and spheres from the M band of 2 wt % agar gel of pH 8.5. The spherulites are lighter than the spheres and show an equatorial notch. (right) Image of these aggregates under crossed polarizers showing interference patterns.

The calcium oxalate aggregates isolated within 3 days from the M band of 2 wt % agar gel of pH 8.5 were COM “spherulites” and “spheres”. The spherulites have an equatorial notch while the spheres do not. Both these aggregates appeared brown in color which is assumed to be caused by the presence of agar in them (Figure 11). The wt % of organic component in the COM spherulites and dumbbells formed in the agar gel of pH 8.5 determined from thermogravimetric analyses is calculated to be 1.5 (see Figures S3–S6).

The spheres appeared darker than the spherulites, an observation which is correlated due to the presence of large amounts of organics in between the crystallite layers compared with spherulites. All these aggregates showed interference patterns under crossed polarizers in an optical microscope (Figure 11).

**Figure 12:**
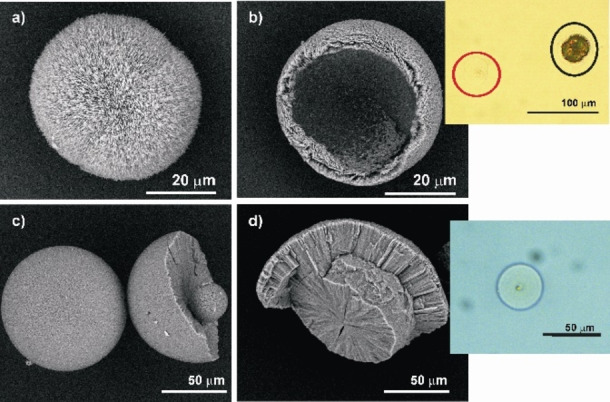
SEM images of COM spherulite formed in the M band of 2 wt % agar gel of pH 8.5 before (a) and after (b) partial decalcification with 0.25 N EDTA. The optical microscopic image of the spherulites in EDTA solution under crossed polarizer is shown in the inset of figure b. The spherulite in red circle is completely decalcified and shows no interference pattern. The spherulite in black circle is only partially decalcified and hence shows interference pattern. Similarly, SEM images of COM spheres grown in the M band of 2 wt % agar gel of pH 8.5 before (c) and after (d) partial decalcification with 0.25 N EDTA. The optical microscope image of the sphere after complete decalcification with the residual agar ghost is shown in the inset of Figure (d).

To determine whether organic material is present inside the crystallite layers, the COM spherulites (Figure 12a) and spheres (Figure 12c) obtained from the M band of 2 wt % agar gels were decalcified with 0.25 N EDTA (ethylenediaminetetraacetate) (see Figures S7 and S8). The demineralization caused a residue consisting of the EDTA insoluble organic material which maintained the shape but without any interference pattern (Insets of figures 12b and d). This is because the decalcified residue is isotropic. The organic material is just an inclusion and there is no orientation correlation between the organic and inorganic material. From the partly decalcified residue of the spherulite, the random orientation of the individual platy crystallites is observed (Figure 12b). It clearly indicates a softer spherical inner region and an outer layer of columnar crystallites (Figure 12d).

The COM spheres generated in agar gel and spherical biogenic calculi exhibit good correspondence.[^3^, ^4^, ^7^] Radial striations and concentric laminations define the spherical COM urinary concretions. Supersaturation, which typically exists in the kidney, results in COM spheres with radial striations.[^38^, ^39^] The primary aggregation of crystallites forming stones and the nucleation of additional crystals on a mucoprotein layer that partially covers their surfaces combine to generate the spherical COM stones.^[40]^ The mucinous layer is absorbed into the crystalline substance as the stones continue to develop, producing the calculus‘s core.^[41]^ Further crystallite nucleation on the core produces radially oriented sheet‐like crystallites that surround the core like a shell. But the precise part played by organic matter in the development of calcium oxalate stones is still unclear. The organic material may participate in epitaxy or may serve as a cementing agent where epitaxy is not a factor. Recently a mapping of the polarity of surfaces of calcium oxalate‐calcium phosphate aggregates using SPEM (Scanning Pyro Electric Microscopy) measurements proposed the mechanism as molecular recognition leading to polar ordering of macromolecules.^[42]^

Twinned COM crystals with dominant {−101} faces were formed in the C band of 2 wt % agar gel of pH 8.5 (Figure 2). These crystals were lighter in colour when compared to the darker spherulites which might be a sign that they contain less organic material in them and hence they are less aggregated. Unlike the rectangular crystallites with no apical faces at all, these crystals have curved apical faces. This is a sign that the apical faces are growing more slowly. In other words, the C band exhibits lower supersaturation than the other bands, which promotes the growth of larger crystals. Additionally, there must be less agar adsorption on the crystal faces, which creates the appearance of apical planes.

**Figure 13:**
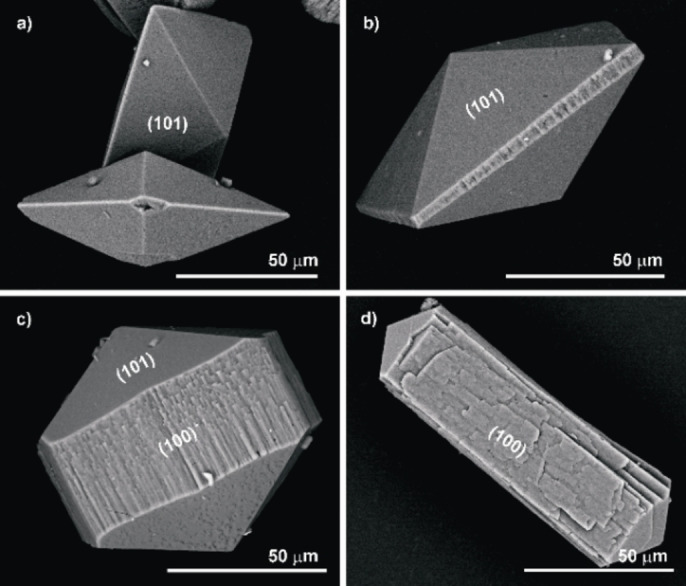
SEM images of COD crystals formed in 2 wt % agar gel of pH 11.5. (a) Tetragonal bipyramids. (b) Tetragonal bipyramidal‐prisms with a combination of {101} and {100} faces. (c) Elongated tetragonal bipyramidal prism. (d) Tetragonal prism. All these aggregates show preferential structuring on the {100} faces

**Figure 14:**
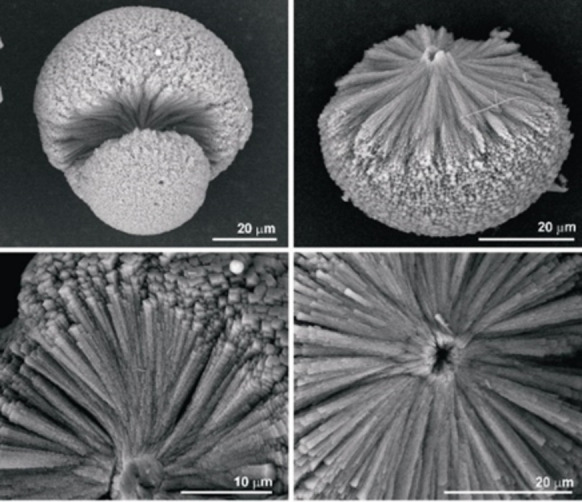
SEM images of the cross‐section of the COD dumbbell composed of rod‐like tetragonal prisms obtained from 2 wt % agar gel of pH 11.5 at 37 °C.

COD crystals were formed when 2 wt % agar gel was used at pH 11.5 under otherwise identical conditions, (Figure 2). Over the course of three days, the aggregates were dispersed throughout the gel. Tetragonal bipyramidal and tetragonal bipyramidal‐prismatic COD made up the area near the calcium source (Figure 2, 13). A region near the oxalate reservoir contained COD aggregates in the form of dumbbells. (Figure 2, 14).

While tetragonal bipyramidal‐prisms have dominant {100} faces, tetragonal bipyramids have dominant {101} faces. The {101} bipyramidal faces appear relatively smooth in all the crystals while structuring is evident on the prism faces. A general principle is that when organic additives are present, COD develops a prismatic habit in which the macromolecule adsorbs on the faces of the {100} prism, inhibiting their growth.^[43]^ As a result, crystal develops along $\left\langle 001\right\rangle$ , where crystal growth is least constrained.

The SEM images of the dumbbells are formed by the “non‐crystallographic” branching and composed by rod‐like tetragonal prismatic COD subindividuals. The broken dumbbells show a cross‐section of a central seed crystal (Figure 14). Magnified SEM images from the broken dumbbells indicate that each half dumbbell is composed of tiny (~micron diameter) rod‐like tetragonal prisms (Figure 14).

From the above results, it is apparent that an increase in the pH value of agar suppresses the growth of COM and favors the growth of COD. Summing up the results obtained in 2 wt % agar gel of pH 8.5, the first crystals formed are spherulites of COM. This is followed by the formation of dumbbells and sheaf of wheat morphologies of COM in the CM band. The sequence ends with formation of twinned COM crystals in the C band. Generally, the highly branched ones occupy the region close to the oxalate source and the COM twins occupy the region close to the calcium source. All the intermediate morphologies are spread between these two extremes. Appearance of kinetically favored COD (dendrites) among the aggregates formed in the M band suggests a high supersaturation attained by the system in this region. It is recognized that at high supersaturations, metastable forms are kinetically favored.^[44]^ The surface adsorption of agar is evident from the change in COM crystal habit from six‐sided platelet to rectangular platelets and needles. Similarly, in the case of 2 wt % agar gel of pH 11.5, dumbbells were formed towards the oxalate source and tetragonal bipyramidal prismatic crystals towards the calcium source.

Spherulites typically are characterized by fibres that radiate outward from a common center. According to classical crystallization, polyhedral crystals bound by flat faces are formed if the crystal growth occurs near equilibrium at low supersaturation and spherulites at higher supersaturations.[^45^, ^46^] A phenomenological theory has been proposed that impurity‐rich layers around the crystallization front cause fibrillation of crystallites and deflection of the tip of the fibres resulting in spherulites.^[45]^

Even though the calcium oxalate stones appear spherulitic; the individual crystallites are plate‐like rather than fibre‐like. The recent findings on the morphology of biogenic stones confirm this.[^8^, ^36^] According to Branda et al., an organic material layer partially covers the (100) face of a crystallite, and the places on that face that are free of organic material act as seeds for the epitaxial formation of a new crystallite layer with the same orientation as the parent crystal.^[47]^ As a result crystalline channels (mineral bridges) are present joining neighboring crystalline layers.^[48]^ Without further investigations, it is intricate to attempt to discuss the prominence and details of “non‐crystallographic” branching mechanism of formation of spherulites of COM and COD in the agar gel system.

The gradients in pH, concentration and super‐saturation are produced as a result of ions diffusing across the gel. From the morphology and location of the aggregates, it becomes apparent that calcium oxalate supersaturation increases on moving from the C band to the O band. Therefore the level of metastability is raised^[47]^ which explains the appearance of otherwise metastable COD in the regions close to the oxalate source. Progressive decrease in supersaturation on moving from the O to the C band generates the various morphologies of COM.

At pH 11.5, the factors favouring the formation of COM are suppressed and hence COD is favoured. This implies that the inhibition of COM by agar reaches a maximum at pH 11.5. A striking relationship between the medium viscosity and the phase that formed has been noted. Fragility of the gel was observed with increase in pH and ions diffuse through high pH agar gels more quickly. COM was crystallized in rigid gels (pH 5) and COD in fragile gels. Also, at pH 5, COM crystals formed towards the middle of the gel while at pH 11.5, the COD aggregates are formed more towards the oxalate source. A higher availability of oxalate leads to crystallization of COD. The adsorption of agar on the crystal faces of COD is evident from the structuring seen on the (100) faces of COD (Figure 13). Agar may be decreasing the interfacial energy of {100} faces of COD. As a result, the otherwise metastable COD nuclei are not being dissolved, and agar retards the growth of COM by adsorbing on the active growth sites. We therefore support the fact that the surface adsorption of agar (impurity effects) together with supersaturation variations causes the morphological variations in this case.^[48]^

COT was not found during our experiments, and it is also only rarely found in urine and kidney stones. This model is relevant for simulating the growth and morphology of urinary stones as urinary stone formation (including nucleation, growth and aggregation of crystals) takes place in human kidney in a fixed, gel‐like state from a flow of supersaturated urine. Moreover, our results support the findings that calcium phosphate substrates are not required for the renal deposition and other factors such as local supersaturations are involved.[^38^, ^48^, ^49^]

## Conclusions

The present work deals with the biomimetic morphological control of calcium oxalate hydrates using agar gel. The results validate that the double diffusion technique in agar gel is a convenient route to grow calcium oxalate aggregates showing close resemblance to biominerals of kidney/urinary calculi and to study their ontogeny. The map presented in Figure 2 summarizes the results. Agar gel at pH 8.5 was found to grow COM dumbbells and spherulites and at pH 11.5, COD aggregates. To the best of our knowledge, COD dumbbells are not grown using a gel matrix. In our earlier studies it was noted that pure gelatine gel and carrageenan gives COM at all pH values while agar gives COM and COD with variation in pH.^[21]^ This emphasizes the fact that the polysaccharides are actively involved in calcium oxalate stone formation. Hence, agar gel is more similar to the conditions prevailing *in‐vivo*. Also, these results reflect the dual role of agar as mineralization promoter and inhibitor of COM the role of which depends upon the pH. This work constitutes first evidence of the promotional effect of polysaccharides based on the agar gel model on the morphogenesis of calcium oxalates and the morphological similarity of COM crystals to biogenic calculi proposes this as the best single model which helps to mimic the multi‐step processes involved in stone formation. This model also gives the scope to study calcium oxalate crystal inhibition by incorporating therapeutic substrates in the gel.

## Experimental Section

The biomineralization of calcium oxalate was mimicked using a double‐diffusion set‐up according to the procedures reported previously for fluorapatite‐gelatin nanocomposites.^[50]^ It consists of two L‐shaped tubes filled with aqueous solutions of CaCl_2_ ⋅ 2H_2_O (0.033 M, 25 mL, pH 8) and Na_2_C_2_O_4_ (0.1 M, 25 mL, pH 8), respectively. The reservoirs of stock solutions are separated by a horizontal tube (16.5 mm in diameter, 75 mm in length) which is filled with agar gel of pre‐adjusted pH. The gel plug was 30 mm in length and 5 cm^3^ in volume. Agar gel was prepared as follows. The required amount of agar (2 wt %) was added to water at 90 °C. After the solution became clear, 2 N NaOH was added while stirring to obtain the desired pH. The pH values of 2 wt % of agar mainly used in this study were 5.5, 8.5 and 11.5. Commercial agar is a mixture of agarose and agaropectin. Alkaline treatment of agar produces agarose in good amounts. The brown color of the gel at higher pH values is due to the hydrolysis of agar using NaOH at elevated temperatures. The temperature was kept constant at 37 °C during the experiments using a water bath. To isolate the products from the gel, the gel plug was pressed out of the tube and cut into slices consisting of the respective Liesegang bands. The pH of the bands was measured with a Mettler Toledo surface electrode. The Liesegang segments were treated with water and the products were washed five times in hot distilled water, centrifuged and finally dried at 40 °C.

Light microscopy images were taken by using an “Axioplan 2 imaging” microscope from the company Carl‐Zeiss equipped with different interchangeable objectives with magnifications of 5‐, 10‐, 20‐, 50‐ and 100‐times and an eyepiece with 10‐times magnifying power. Pictures were recorded using the software AnalySIS labFlow [Soft Imaging System GmbH, Version 5, Munster].

The phase composition of the samples was determined by X‐ray diffraction analyses. The samples were ground well, suspended in ethanol and mounted on a Kapton film adhering on aluminium rings. X‐ray powder data were collected in transmission mode using a Huber G670 Image Plate Camera, Cu *K*α_1_‐ radiation (λ=1.540598 Å) and a germanium (111) monochromator. Data collections were made in the range of 3°≤2θ≤100° with a step size of 0.005° 2θ (exposure time 90 min). Data manipulation of the X‐ray powder diffraction data was made by using the commercially available STOE WinXPOW [Version 1.2, Programm zur Messung und Auswertung von Röntgenpulverdiffraktogrammen, STOE & Cie GmbH., Darmstadt].

For scanning electron microscopy (SEM), the samples were mounted onto conductive carbon tapes adhered to aluminum or brass holders and were sputter coated with gold for a minute with Cressington108 Auto Sputter coater. The morphology of the aggregates obtained was studied by means of an ESEM FEI Quanta 200 FEGi system operated at a low vacuum (70 Pa) mode and at an acceleration voltage of 15 kV (FEI Company, Eindhoven, NL). High resolution images were taken using a solid‐state back scattered electron (SSD‐BSE) detector.

Transmission electron microscopy (TEM) analysis was carried out on a FEI Tecnai F30‐G2 with Super‐Twin lens (FEI) with a field emission gun at an acceleration voltage of 300 kV. The point resolution amounted to 2.0 Å, and the information limit to about 1.2 Å. The microscope was equipped with a wide‐angle slow scan CCD camera (MultiScan, 2 k×2 k pixels; Gatan Inc., Pleasanton, CA, USA).

## Conflict of Interests

The authors declare no conflict of interest.

1

## Supporting information

As a service to our authors and readers, this journal provides supporting information supplied by the authors. Such materials are peer reviewed and may be re‐organized for online delivery, but are not copy‐edited or typeset. Technical support issues arising from supporting information (other than missing files) should be addressed to the authors.

Supporting Information

## Data Availability

Data sharing is not applicable to this article as no new data were created or analyzed in this study.
