# Supplementary material for: Biomimetic Growth of Calcium Oxalate Hydrates: Shape Development and Structures in Agar Gel Matrices
Source: Chemistry. 2025 Apr 14;31(25):e202404269. doi: 10.1002/chem.202404269 (PMC12057605; doi:10.1002/chem.202404269)
Supplement: Supplementary file 1 — Supporting Information [file CHEM-31-e202404269-s001.pdf]

Chemistry–A European Journal

Supporting Information

Biomimetic Growth of Calcium Oxalate Hydrates: Shape Development and Structures in Agar Gel Matrices

Annu Thomas, Paul Simon,* Wilder Carrillo-Cabrera, and Elena Sturm

Supporting Information

Given below are the pictures of 2 wt.% agar gel at pH 5, 8.5 and 11.5 (Figure S1) and the crystals obtained in it (Figure S2). The gels are stable up to pH 12.5.

pH of 2 wt.% Agar gel	Figure before the diffusion	Figure after the diffusion
5	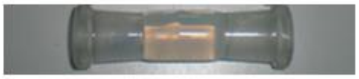	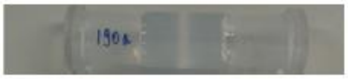
8.5	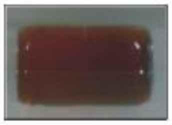	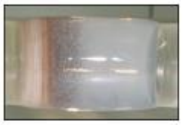
11.5	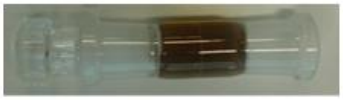	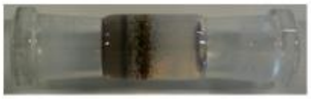

Figure S1. Photograph of 2 wt. % agar at pH values 5, 8.5 and 11.5 before and after the double diffusion.

pH of 2 wt.% Agar gel	Crystals formed in			
	**C band**	**CM band**	**M band**	**O band**
5	No crystals formed	No crystals formed	COM 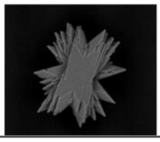	No crystals formed
8.5	COM 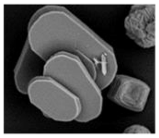	COM 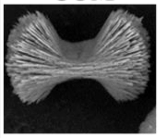	COM 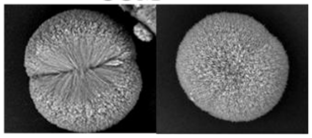	No crystals formed
11.5	COD 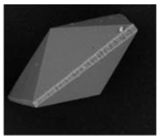	COD 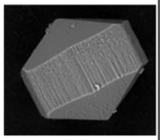	COD 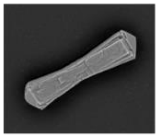	COD 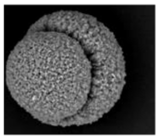

Figure S2. Comparison of crystals grown at pH 5, 8.5, and 11.5. Note that crystals are formed in different bands.

The wt. % of organic component in the COM spherulites and dumbbells formed in agar gel of pH 8.5 was determined from thermogravimetric analyses (Figure S3). It is calculated to be 1.5.

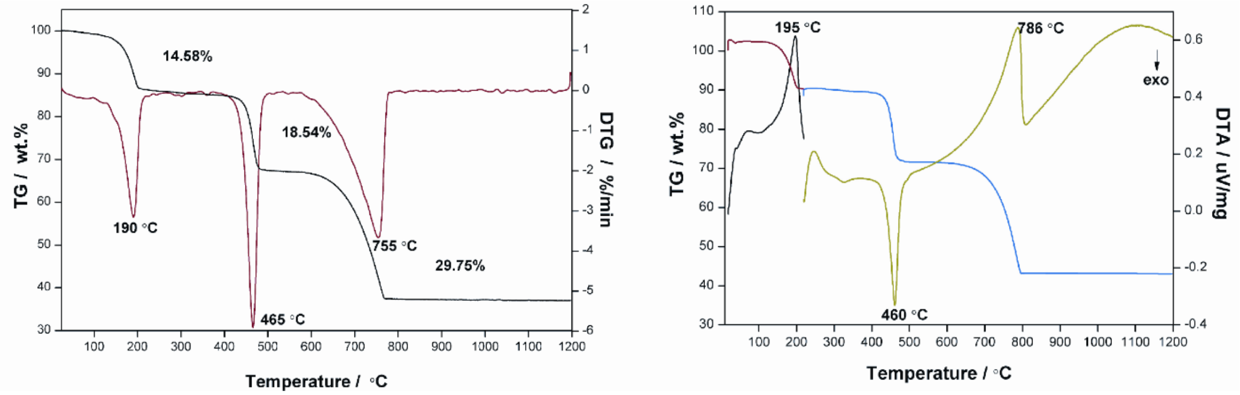

Figure S3. Thermogravimetric analysis of COM from 2wt.% agar gel of pH 8.5.

The decomposition processes of COM dumbbells and spherulites from 2 wt. % agar gel of pH 8.5, were investigated by means of TG/DTA/MS. The sample was ground well and washed many times with distilled water and dried before starting the measurement. The heating was performed in an alumina crucible with a rate of 5 K/min up to 1300 ºC in two steps (Ar and O_2 atmospheres). The TG/DTG/DTA of COM aggregates (Figure S3) clearly indicates stepwise mass losses of 14.58 % (165 – 202 °C), 18.54 % (450 – 476 °C) and 29.75 % (697 -790 °C).

A combined analysis of TG/DTA together with MS ensures the complete removal of water in the first step (Figure S4, (a, m/z = 18)). The endothermic peak in DTA corresponding to release of water appears at 195 °C. The second step which corresponds to an exothermic peak at 460 °C is characterized by the release of minor fragments with molecular masses 12 (C^+), 22 (CO_2^{2+}), 30 (further reaction of CO), 44 (CO_2), 45 ($C_2H_5O^+$) and 46 (further reaction of CO_2) (Figure S4 (b,c)). The final thermal decomposition takes place in the third step which corresponds to an endothermic peak at 786 °C. From the TGA, the wt. % of organic in the sample was calculated to be 1.5. The presence of such small fractions of organic component in the COM aggregates makes the qualitative analysis of organic component difficult even by mass spectroscopy. Therefore, higher molecular mass fragments are not detected in the mass spectrum of COM spherulites in contrast to that of pure agar (Figures S5 and S6).

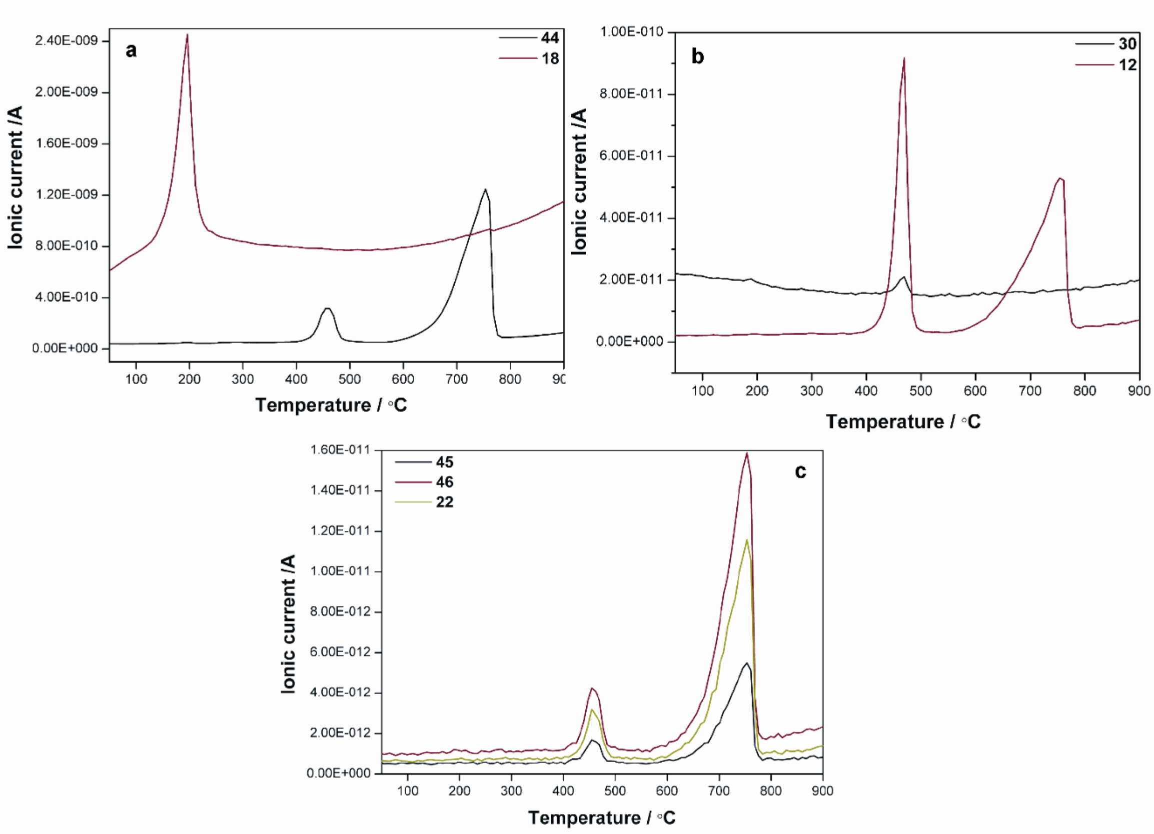

Figure S4**.** Mass spectrum of COM spherulites from 2 % agar gel.

The thermal decomposition of pure agar takes place in two steps at 95°C and 285 °C with mass loss of 5.86 % (72.9 – 126.9 °C) and 65.20% (251 – 445 °C) (Figure S5). Mass spectrometric analysis of the evolved gases shows that water is evolved at 109 °C. The second step (200 to 400 °C) is more complex with the release of main fragments with molecular masses of 18 and 44 corresponding to H_2O and CO_2 respectively (Figure S6 (a,b)). Small amounts of fragments with molecular masses 12, 15, 22, 31, 45, 53, 69 (Figure S6 (c,d)) indicate the release of products of partial decomposition and simultaneous oxidation of agarose (C^+, CH_3^+, CO_2^{2+},CH_3O^+, $C_2H_5O^+$,$C_4H_5^+$, $C_4H_5O^+$).

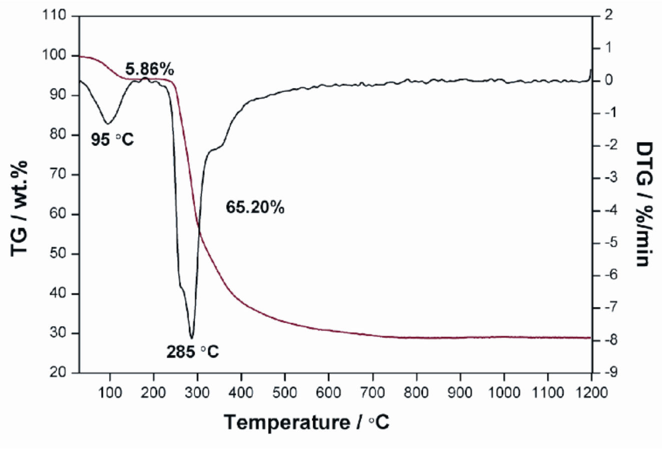

Figure S5. TG/DTG of pure agar.

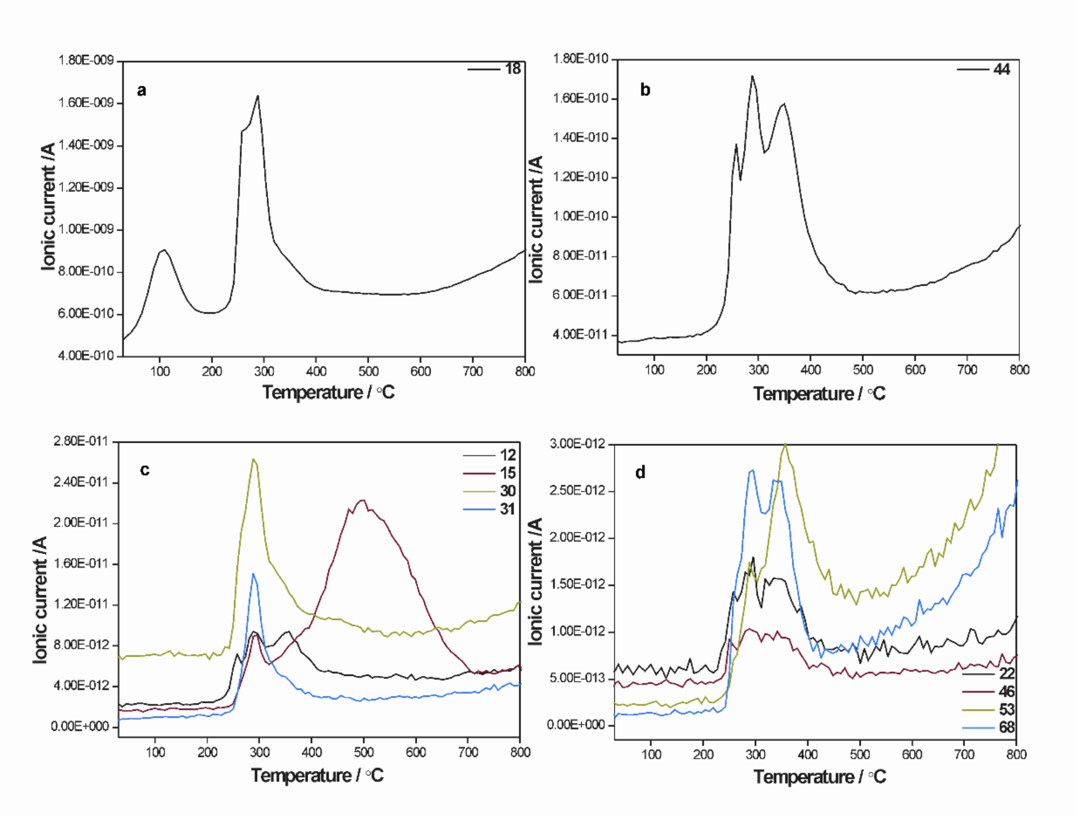

Figure S6. Mass spectrum of pure agar.

Again, in an attempt to check the presence of organic component in these spherulites, both COM and COD spheres were decalcified with 0.25N EDTA (Figures S7 and S8). The decalcification resulted in the retention of the EDTA insoluble organic material (crystal ghost) which maintained the shape. The agar ghosts from COD spheres did not retain the Brewster cross (Figure S8). The demineralization caused a residue consisting of the EDTA insoluble organic material which maintained the shape but without any interference pattern. This is because the decalcified residue is isotropic and the organic material is just an inclusion.

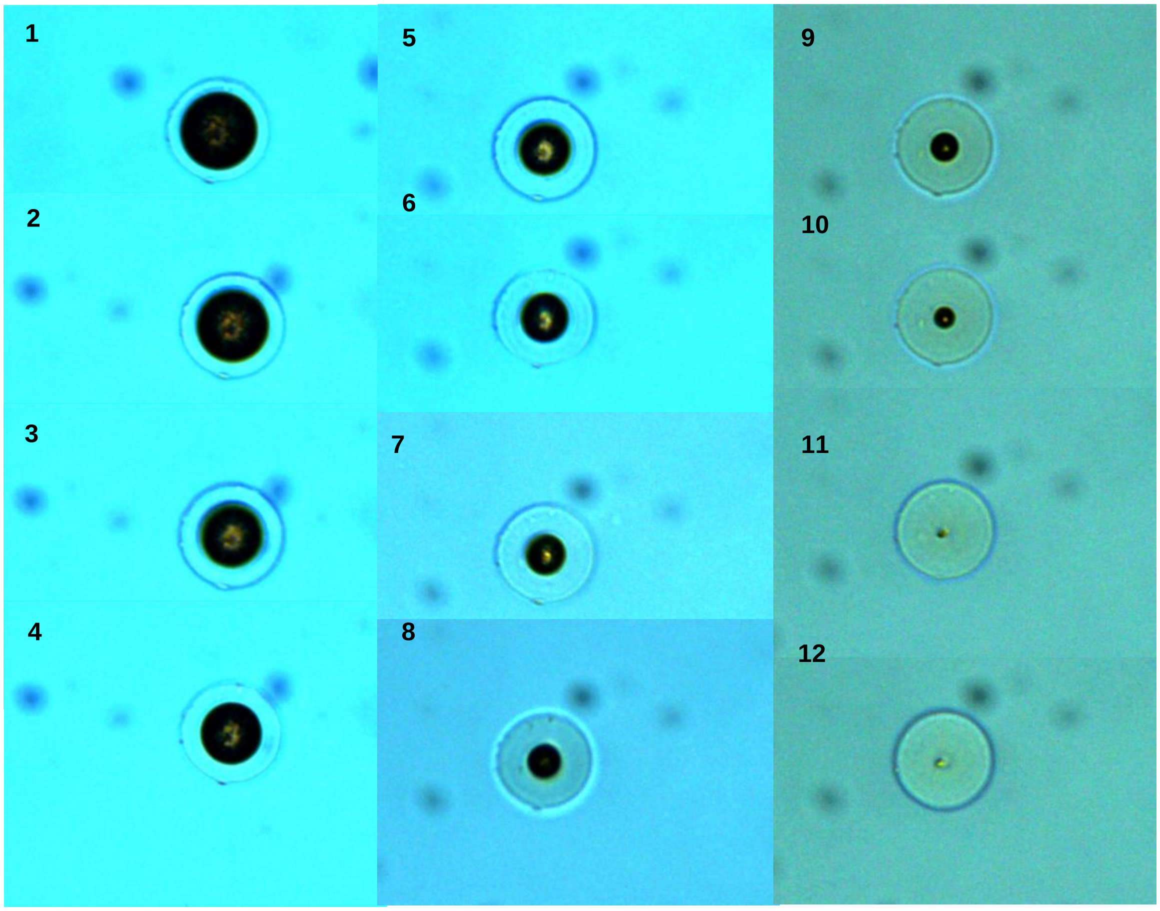

Figure S7. (1-11) Decalcification of COM spheres with EDTA solution. Agar ghost remains in the end (12).

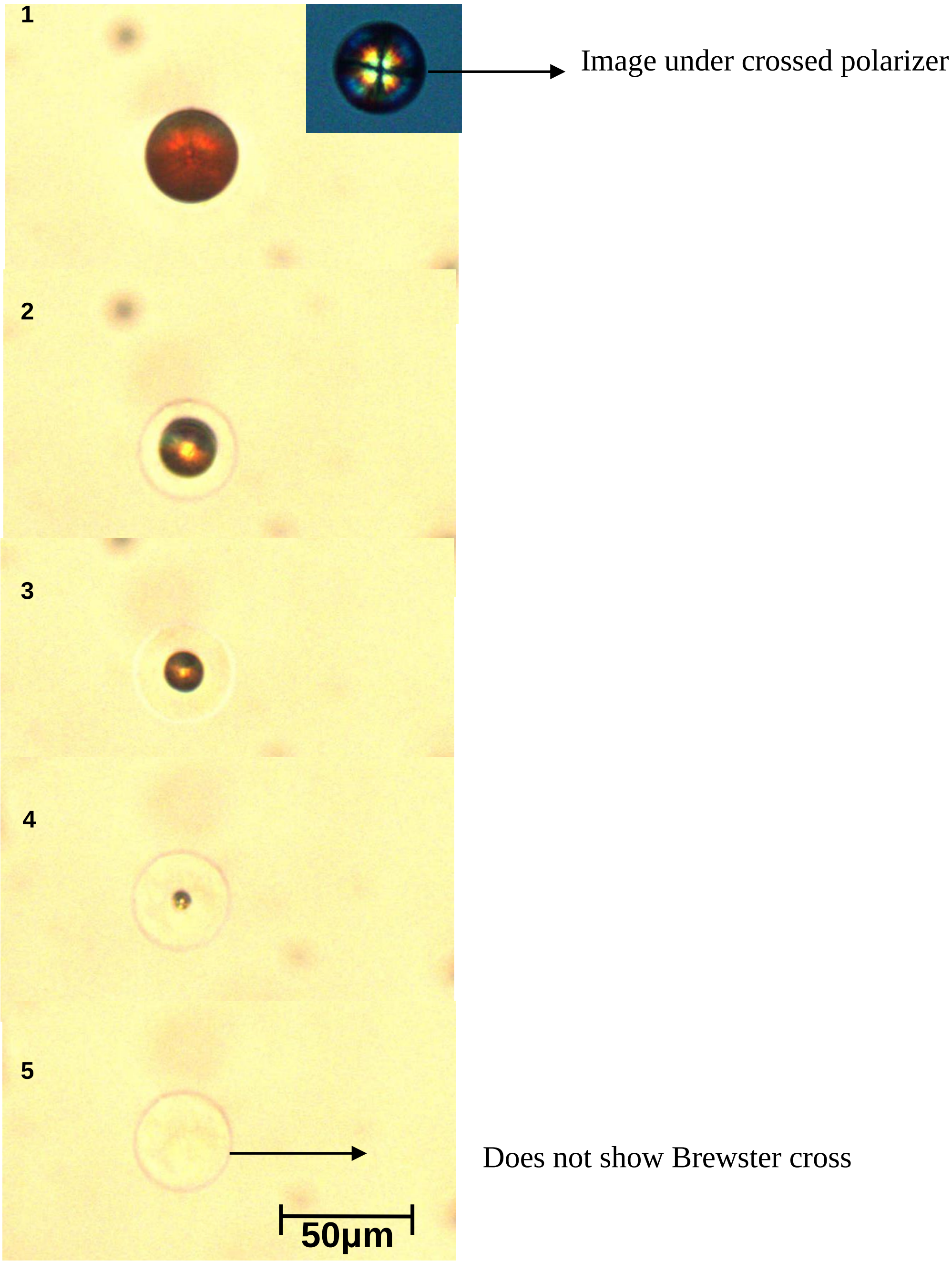

Figure S8. (1-5) Decalcification of COD spheres with EDTA solution. Inset shows the typical Brewster cross of the COD sphere under the crossed polarizer. (5) Agar ghost remaining in the end does not show the Brewster cross.
